# Invasive lobular carcinoma arising in accessory breast tissue

**DOI:** 10.1186/1477-7819-11-47

**Published:** 2013-02-26

**Authors:** Catriona Devine, Carol-Ann Courtney, Rahul Deb, Amit Agrawal

**Affiliations:** 1 Department of Breast Surgery, Royal Derby Hospital, Uttoxeter Road, DE22 3NE, Derby, UK; 2 Department of Pathology, Royal Derby Hospital, Uttoxeter Road, DE22 3NE, Derby, UK; 3 Nottingham Breast Institute, Hucknall Road, NG5 1PB, Nottingham, UK; 4 Department of Breast Surgery, Graduate Entry Medicine (GEM) School, Royal Derby Hospital, University of Nottingham, DE22 3DT, Derby, UK

## Abstract

**Background:**

Lobular carcinoma in accessory breast tissue is a rare occurrence. We present such a case in a 61-year-old woman.

**Case presentation:**

A skin nodule in the axillary skin on excision biopsy revealed invasive lobular carcinoma.

**Conclusions:**

Carcinoma in accessory breast tissue is uncommon especially invasive lobular type. A high index of suspicion may avoid late diagnosis.

## Background

Accessory breast tissue is seen in 2% to 6% of the population [1] with carcinoma in this accessory tissue reported rarely. The most common morphological variant is invasive ductal carcinoma. We present a rare case of invasive lobular carcinoma in the accessory breast tissue.

## Case presentation

A 61-year-old postmenopausal woman with a 14-month history of 3 × 2.5 cm, indurated rubbery nodule of the left axilla was referred by the dermatologist to the plastic surgeons for an excision biopsy with 1 cm margin for a possible soft tissue tumor.

**Figure 1:**
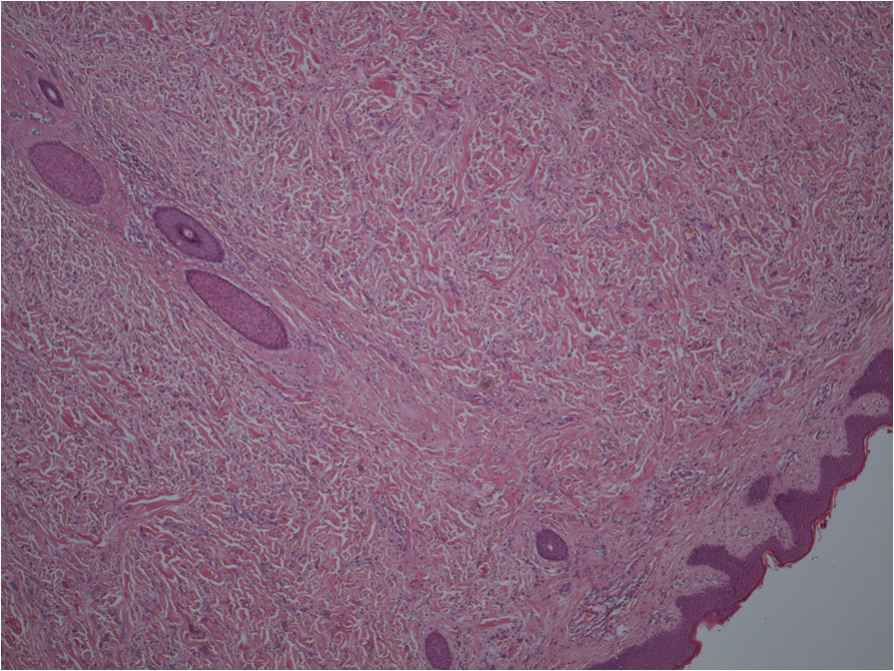
Lobular carcinoma with axillary skin (Magnification ×40).

**Figure 2:**
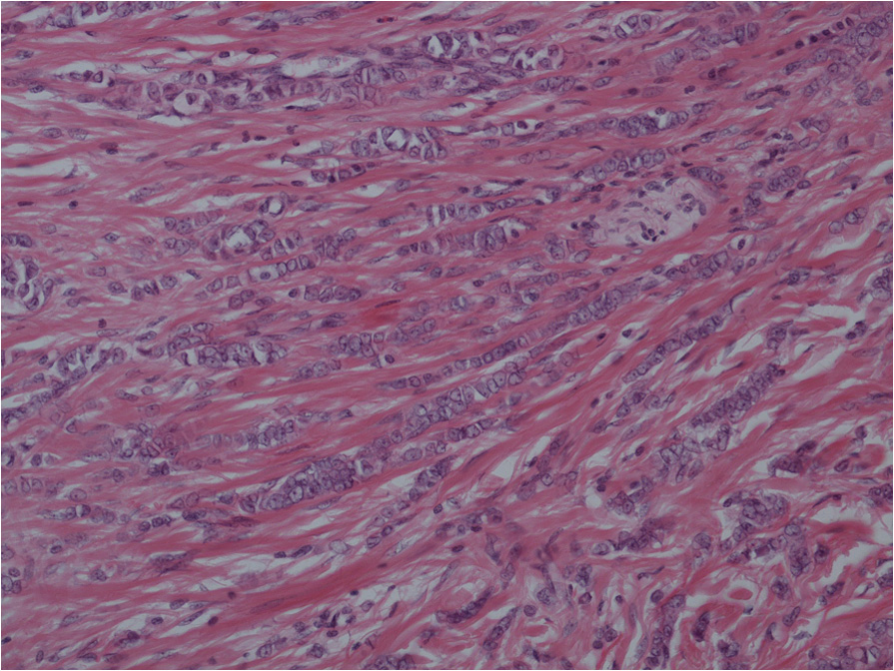
Lobular carcinoma with dermis (Magnification ×200).

Histology revealed an unexpected primary breast cancer: grade 2 invasive lobular carcinoma measuring 2.2 cm on a background of lobular carcinoma *in situ* along with normal breast tissue (Figures 1 and 2). The patient was then referred to the breast multidisciplinary team (MDT) for further management.

There was no previous personal or family history of breast cancer. Subsequent mammography and breast magnetic resonance imaging (MRI) were reported as normal. As the margins were involved she underwent re-excision of margins with axillary node sampling. Both the new margins and the nodes were free of disease.

The patient underwent adjuvant radiotherapy to the breast and was commenced on adjuvant anastrozole for this estrogen receptor positive tumor.

## Conclusions

The incidence of accessory breast is 2% to 6% of the general population [1]. It is the consequence of partial regression of the primitive milk streak which forms in the human embryo [2,3]. Accessory breast tissue is seen along the milk line [4] but is most frequent in the axillary region.

Embryologically being breast tissue, the accessory breast tissue is subject to homeostatic hormonal controls too and thus may become clinically apparent during puberty or pregnancy. Similarly, it is also subject to pathological changes that occur in the normal anatomical site of the breasts. There are numerous reports of masses arising in accessory breast tissue including fibroadenomas and breast cancers [1,2,5,6]. The principal malignancy identified in accessory breast tissue, as with normal breasts, is invasive ductal carcinoma (79%), followed by medullary and lobular carcinomas which are seen in less than 10% of cases [6]. Accessory axillary carcinoma is a rare form of breast cancer. In this case report, the patient had both invasive lobular carcinoma and lobular carcinoma *in situ* in the accessory axillary tissue, which is an unusual finding.

This case report presents an invasive carcinoma discovered early with no lymph node involvement. The overall prognosis is similar to carcinoma of normal breast in the same tumor, node, metastasis stage, although given the location within the axillary lymph node basin, the likelihood of metastases is high [7]. It is, therefore, imperative that a lump in the axillary region is triple assessed as in any breast pathology to rule out carcinoma in the accessory axillary tissue to achieve a potentially curable status. It is also important to evaluate for accessory tissue on the contra-lateral side because 13% of the cases are bilateral in normal breast [8].

The standard UK practice is to perform MRI of the breasts in suspected or diagnosed mammographically occult invasive lobular cancer and, therefore, this imaging modality should be used if there is a high index of suspicion of carcinoma in accessory breast tissue [9]. Adjuvant systemic therapy should be guided by the standard guidelines and practice (such as according to estrogen receptor (ER), human epidermal growth factor receptor-2 (HER2) status, tumor grade, stage, prognostic indices) [6,10].

## Consent

Written informed consent was obtained from the patient for publication of this Case report and any accompanying images.

## Competing interests

The authors declare that they have no competing interests.

## Authors’ contributions

CD drafted the manuscript. CAC was clinically responsible for the patient’s care. RD was responsible for pathology. AA was responsible for initiation of the study and revised the manuscript. All authors read and approved the final manuscript.
